# Supplementary material for: Location bias contributes to functionally selective responses of biased CXCR3 agonists
Source: Nat Commun. 2022 Oct 4;13:5846. doi: 10.1038/s41467-022-33569-2 (PMC9532441; doi:10.1038/s41467-022-33569-2)
Supplement: Supplementary file 1 — Supplementary Information [file 41467_2022_33569_MOESM1_ESM.docx]

**SUPPLEMENTAL INFORMATION**

**TITLE:**

Location bias contributes to functionally selective responses of biased CXCR3 agonists

**AUTHORS:**

Dylan Scott Eiger^1^

Noelia Boldizsar^2^

Christopher Cole Honeycutt^2^

Julia Gardner^2^

Stephen Kirchner^3,4^

Chloe Hicks^2^

Issac Choi^5^

Uyen Pham^1^

Kevin Zheng^6^

Anmol Warman^2^

Jeffrey S. Smith^6-10^

Jennifer Y. Zhang^3, 11^

Sudarshan Rajagopal^1,5,*^

^*^ = corresponding author

**AFFILIATIONS:**

^1^Department of Biochemistry, Duke University, Durham, NC, 27710, USA

^2^Trinity College, Duke University, Durham, NC, 27710, USA

^3^Department of Dermatology, Duke University, Durham, NC, 27707, USA

^4^Department of Molecular Genetics and Microbiology, Duke University, Durham, NC, 27707, USA

^5^Department of Medicine, Duke University, Durham, NC, 27710, USA

^6^Harvard Medical School, Boston, MA, 02115, USA

^7^Department of Dermatology, Brigham and Women’s Hospital, Boston, MA, 02115, USA

^8^Department of Dermatology, Beth Israel Deaconess Medical Center, Boston, MA, 02215, USA

^9^Dermatology Program, Boston Children’s Hospital, Boston, MA, 02115, USA

^10^Department of Dermatology, Massachusetts General Hospital, Boston, MA, 02114, USA

^11^Department of Pathology, Duke University, Durham, NC, 27710, USA

**CORRESPONDENCE**

[sudarshan.rajagopal@duke.edu](mailto:sudarshan.rajagopal@duke.edu)

Supplementary Information:

- **Supplementary Figure 1:** Raw luminescence values of KB-1753-nLuc constructs and alternative data normalization. Related to Figure 2.
- **Supplementary Figure 2:** Gai mediated cAMP inhibition at CXCR3 as measured using a nuclear localized cAMP sensor. Related to Figure 3.
- **Supplementary Figure 3:** Plot of maximal G protein and b-arrestin signaling at different subcellular locations. Related to Figure 4.
- **Supplementary Figure 4:** ERK activation at 30 and 60 minutes and premise of ERK biosensor. Related to Figure 5.
- **Supplementary Figure 5:** Approach and source data for RNA-seq to assess transcription in CD8+ T cells. Related to Figure 6.
- **Supplementary Table 1:** Key resources and DNA constructs used in this study.

**
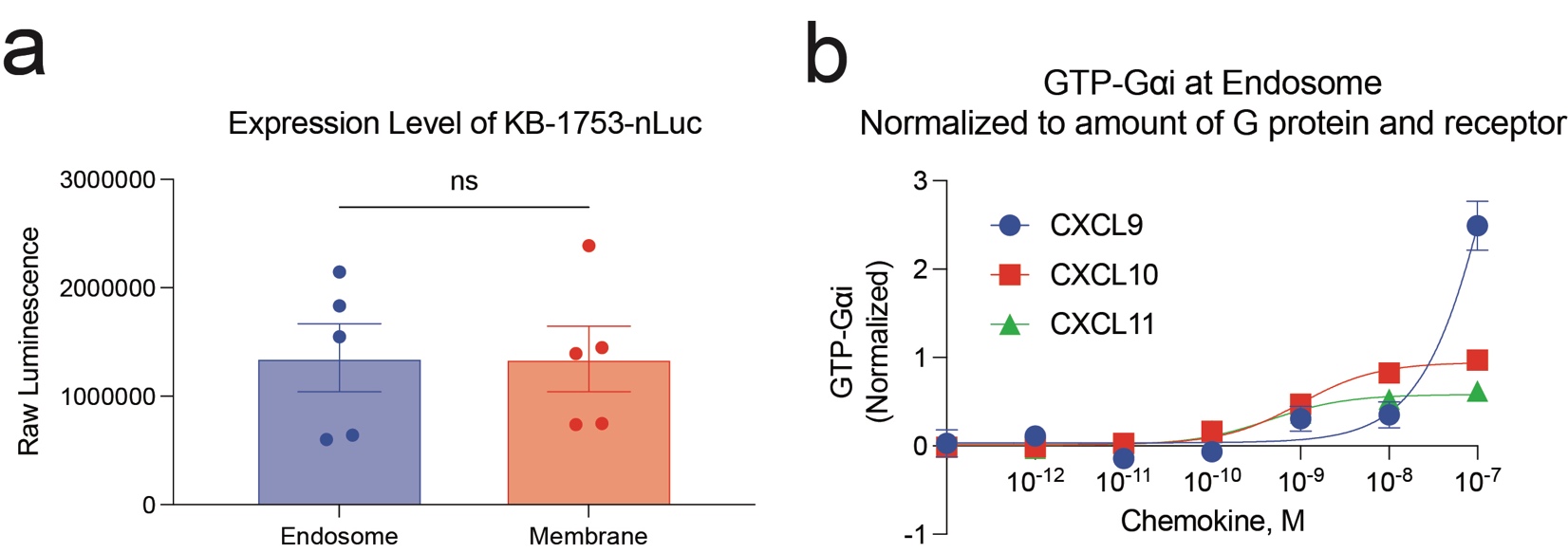
**

**Supplementary Figure 1: Raw luminescence values of KB-1753-nLuc constructs and alternative data normalization. Related to Figure 2**. (A) Raw luminescence values of the KB-1753-nLuc constructs at the endosome and the plasma membrane. Data are the mean ± SEM, n = 5 independent plate-based experiments. ns P ≥.05. denotes statistically significant differences between paired luminescence averages between the location specific nanoluciferase construct as measured using a two-sided paired t-test. (B) Alternative normalization approach for assessing G protein activation. Specifically, the amount of active G protein (Figure 2c-d) normalized to maximum signal was divided by the amount of total G protein (Figure 2i-j) normalized to maximum signal which was further divided by the amount of receptor present in endosomes (Figure 1a) normalized to maximum signal.

**
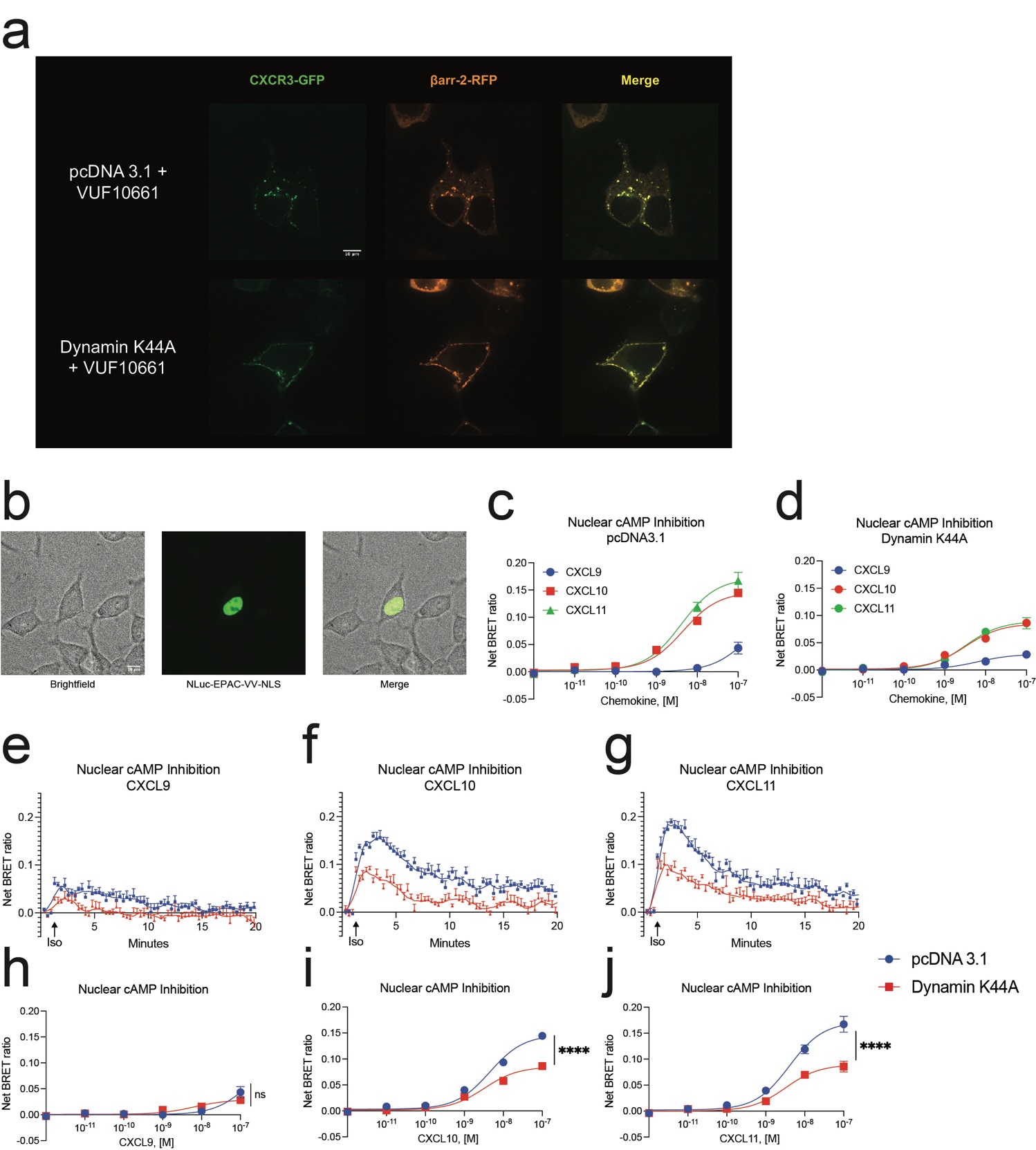
**

**Supplementary Figure 2: Gαi mediated cAMP inhibition at CXCR3 as measured using a nuclear localized cAMP sensor. Related to Figure 3. (a)** Confocal microscopy images of HEK293 cells transfected with CXCR3-GFP, β-arrestin 2-RFP (β-arr-2-RFP), and either pcDNA 3.1 or Dynamin K44A demonstrating successful inhibition of endocytosis with overexpression of Dynamin K44A. following stimulation with 1µM VUF10661 for 45 minutes **(b)** Confocal and brightfield microscopy images of HEK293 cells transfected with a nuclear localized cAMP BRET biosensor (NLuc-EPAC-VV-NLS). Agonist dose-dependent inhibition of isoproterenol-induced nuclear cAMP production by the chemokine in HEK293 cells with concurrent transfection of **(c)** pcDNA 3.1 or **(d)** Dynamin K44A to inhibit internalization. **(e to g)** Kinetic data (100nM chemokine) and **(h to j)** agonist dose-dependent inhibition of cAMP signal, as measured between 3- and 5-minutes, in HEK293 cells treated with chemokine. Data are the mean ± SEM, n = 5 independent plate-based experiments. Extra sum of squares F test was used for **(h-j)** ns P ≥.05, * P 0.01-0.05, ** P 0.001-0.01, *** P 0.0001 to 0.001, **** P < 0.0001 denotes statistically significant differences between E_max_ for dose response data of pcDNA 3.1 versus Dynamin K44A transfection conditions at each ligand.

**
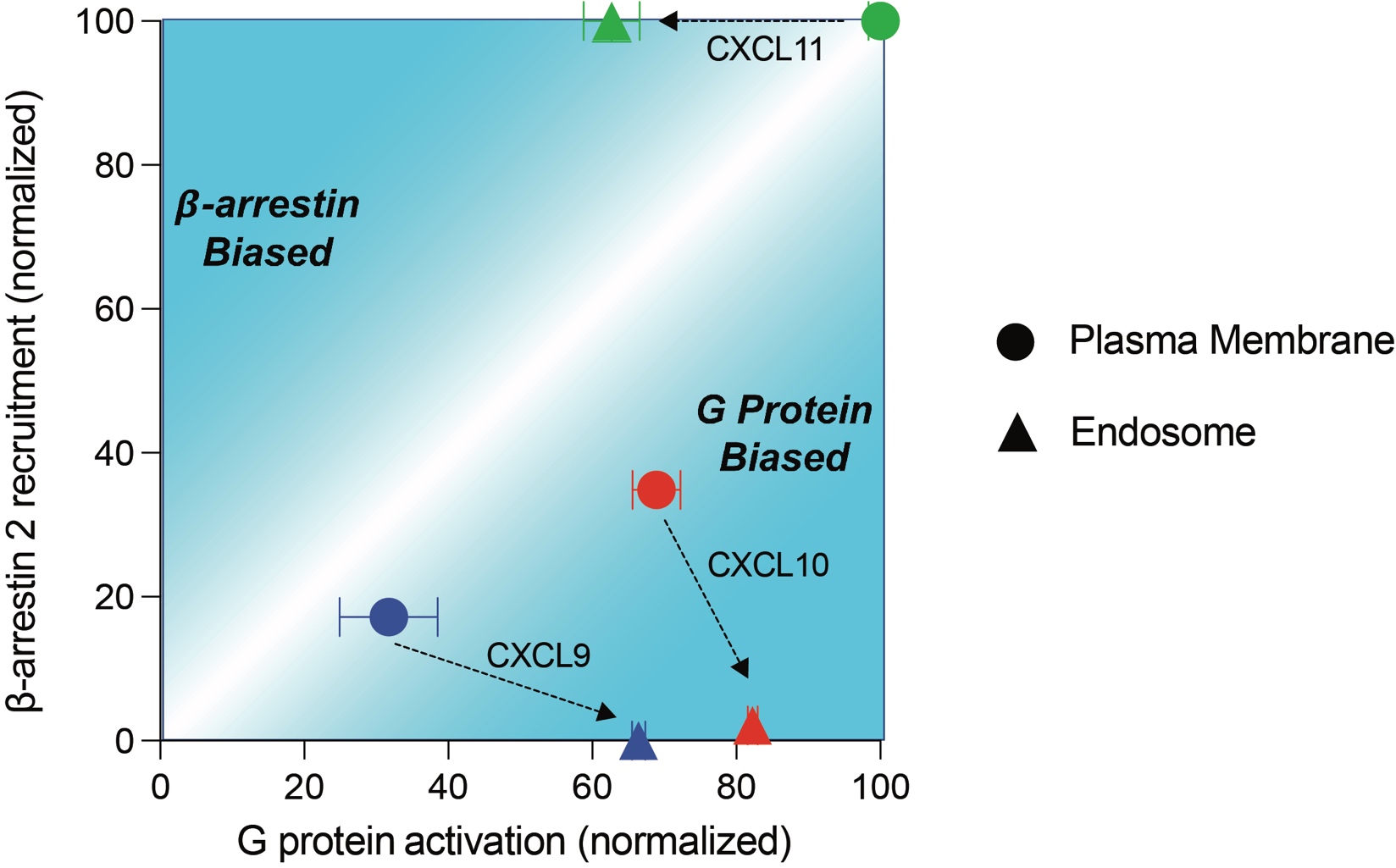
**

**Supplementary Figure 3: Plot of maximal G protein and β-arrestin signaling at different subcellular locations. Related to Figure 4.** Plot of maximal G protein activation and β-arrestin 2 recruitment at the plasma membrane and endosome. All data are normalized to the values for CXCL11 at the plasma membrane. Gαi activation at the endosome was calculated by dividing the relative amount of endosomal Gαi-GTP by total endosomal Gαi. Data shown are derived from Figures 2 and 3. Specifically, mean values and error bars of G protein activation are determined by taking maximum Gαi-GTP for each chemokine from Figure 2e-g and dividing this data by total maximal GαI for each chemokine from figure 2i. Total GαI at the plasma membrane is assumed to be the same for each chemokine. For β-arrestin 2 recruitment, mean values and error bars are obtained from figures 4b and 4d.

**
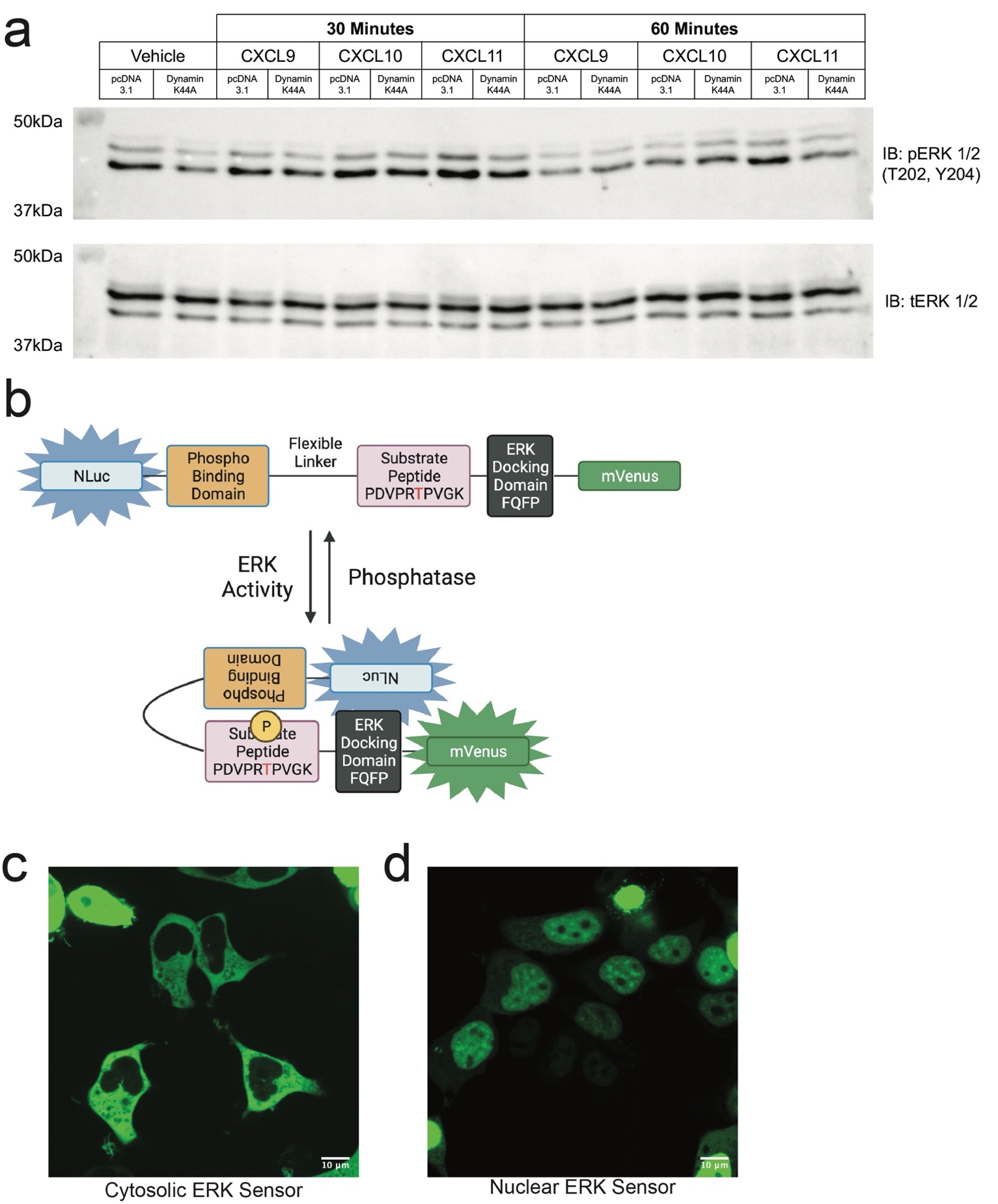
**

**Supplementary Figure 4: ERK activation at 30 and 60 minutes and premise of ERK biosensor. Related to Figure 5. (a)** Representative immunoblot of n=5 independent experiments of ERK1/2 phosphorylation following 30 and 60 minutes of stimulation with vehicle control or 100nM of chemokine with transfection of pcDNA 3.1 or Dynamin K44A. **(b)** Schematic of the BRET based ERK biosensor^58^. The biosensor consists of an N-terminal nanoluciferase (NLuc), phosphobinding domain, flexible linker, ERK substrate peptide, ERK docking domain, and C-terminal mVenus. Following phosphorylation of the target peptide by activated ERK, the phosphobinding domain will complex with the phosphothreonine, bringing the NLuc and mVenus in close proximity to generate a BRET signal. **(c and d)** Confocal microscopy of the ERK biosensors targeted to the cytoplasm or the nucleus. Images are representative of three independent replicates.

**
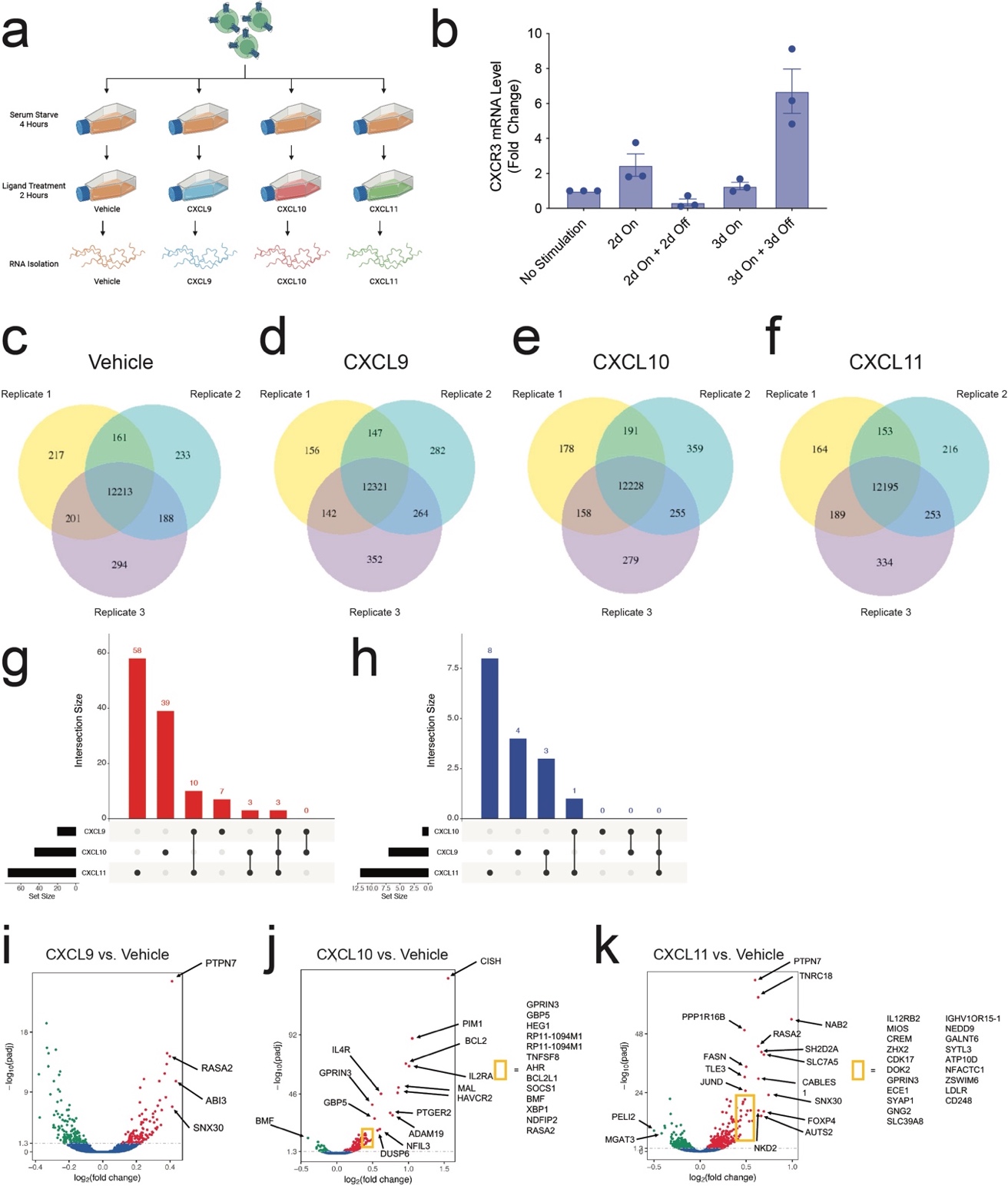
**

**Supplementary Figure 5: Approach and source data for RNA-seq to assess transcription in CD8+ T cells. Related to Figure 6. (a)** Schematic of experimental design of RNA-seq experiments on activated primary CD8+ T cells. T cells were cultured with anti-CD3/CD28 T-cell Dynabeads (Thermo Fisher) for three days and then three days without Dynabeads to induce T cell activation and expansion. T cells were serum starved for four hours and incubated with the listed treatment condition (100nM) for 2 hours. RNA was then isolated and then processed for RNA sequencing. **(b)** Quantitative PCR (qPCR) of peripheral blood mononuclear cells to examine CXCR3 transcript levels following stimulation with anti-CD3/CD28 magnetic beads. Cells were cultured under five conditions: No stimulation, 2 days with magnetic beads (2d On), 2 days with magnetic beads followed by 2 days without magnetic beads (2d On + 2d Off), 3 days with magnetic beads (3d On), or 3 days with magnetic beads followed by 3 days without magnetic beads (3d On + 3d Off). Transcript levels were normalized using 18s rRNA, and then subsequently normalized to the No stimulation condition. Data are the mean ± SEM, n=3 independent experiments carried across different days using the same primary donor cells. **(c-f)** Venn diagram showing common transcripts identified across three replicates within each treatment group demonstrating high degrees of replicability between replicate samples. UpSet Plots demonstrating similarly or differentially **(g)** upregulated or **(h)** downregulated transcripts at a Log2(Fold Change) of >±0.3. The UpSet Plots demonstrate that the majority of differential gene expression observed in our data set is not shared between the chemokines. **(i-k)** Volcano plots comparing differentially expressed transcripts between the listed treatment condition and vehicle control. Labelled transcripts are statistically significant and demonstrate a Log2(Fold Change) of >±0.4. P-values were adjusted using the Benjamini and Hochberg’s approach for controlling the False Discovery Rate (FDR).

**
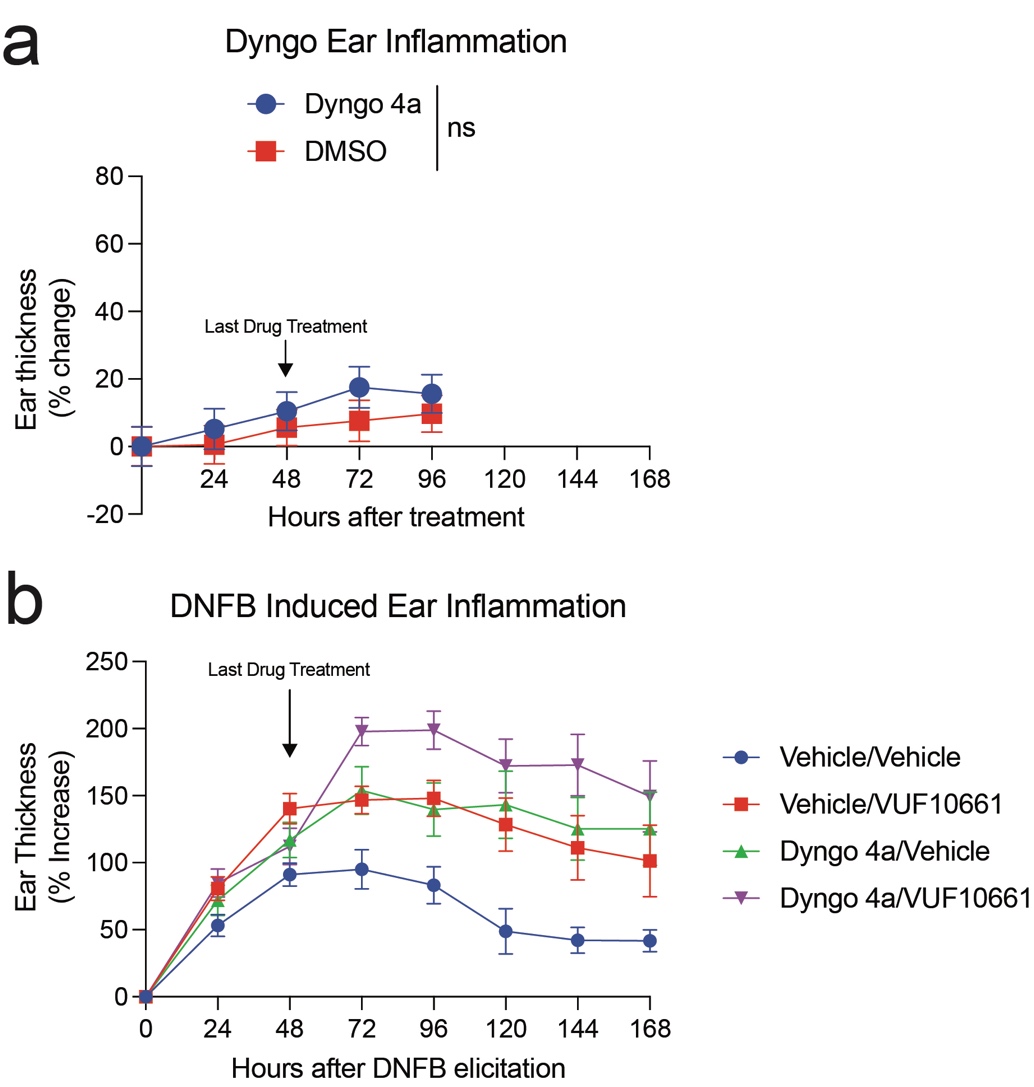
**

**Supplementary Figure 6: Dyngo 4a treatment alone does not illicit an inflammatory response and raw data for each mouse group. Related to Figure 7. (A)** Ear thickness following application of Dyngo 4a (50nM) or DMSO control in the absence of VUF10661 or dinitrofluorobenzene (DNFB) to assess for a nonspecific effect of Dyngo 4a treatment. Mice were treated with listed treatments at 0 hours, 24 hours, and 48 hours**.** Data are means ± SEM of 8 mice per treatment group. ns P ≥.05, * P 0.01-0.05, ** P 0.001-0.01, *** P 0.0001 to 0.001, **** P < 0.0001 using a two-way ANOVA analysis. **(B)** Raw increase in ear thickness following DNFB induced contact hypersensitivity for two experimental groups (Vehicle/VUF10661 and Dyngo 4a/VUF10661) and two control groups (Vehicle/Vehicle and Dyngo 4a/Vehicle). Data are means ± SEM, n=8 for vehicle/vehicle, n=9 for vehicle/VUF10661, n=7 for Dyngo/Vehicle, and n=7 for Dyngo/VUF10661.

**Supplementary Table 1: Key resources and DNA constructs used in this study.**

| **RESOURCE** | **SOURCE** | **IDENTIFICATION** |
| --- | --- | --- |
| **Antibodies** | | |
| Donkey polyclonal anti-rabbit IgG peroxidase conjugated | Rockland | Cat#611-7302 |
| Sheep polyclonal anti-mouse IgG peroxidase conjugated | Rockland | Cat#610-603-002 |
| Mouse monoclonal anti-phospho-p44/42 MAPK 1/2 (ERK1/2) (Thr202/Tyr204) | Cell Signaling Technologies | Cat#9106 |
| Rabbit polyclonal anti-MAPK 1/2 (ERK1/2) | Millipore Sigma | Cat#06-182 |
| **Bacterial Strains** | | |
| XL10-Gold Ultracompetent E. Coli | Agilent | Cat#200315 |
| **Chemicals, peptides, and recombinant proteins** | | |
| Recombinant Human CXCL9 | Peprotech | Cat#300-26 |
| Recombinant Human CXCL10 | Peprotech | Cat#300-12 |
| Recombinant Human CXCL11 | Peprotech | Cat#300-46 |
| VUF10661 | Sigma-Aldrich | Cat#SML0803 |
| Dyngo 4a | Abcam | Cat#AB120689 |
| 1-Fluoro-2,4-dinitrobenzene | Sigma-Aldrich | Cat#D1529 |
| GlutaMax | Gibco | Cat#35050061 |
| Antibiotic-Antimycotic | Gibco | Cat#15240062 |
| FlAsH-EDT2 | Santa Cruz Biotechnology | Cat#sc-363644 |
| Coelenterazine h | Cayman Chemical | Cat#16894 |
| Coelenterazine h | NanoLight Technology | Cat#301 |
| QuikChange Lightning Site-Directed Mutagenesis Kit | Agilent | Cat#210518 |
| PhosSTOP | Sigma-Aldrich | Cat#4906845001 |
| cOmplete Protease Inhibitor Cocktail | Sigma-Aldrich | Cat#11697498001 |
| SuperSignal West Pico PLUS Chemiluminescent Substrate | Thermo Fischer Scientific | Cat#34580 |
| Dynabead Human T-Activator CD3/CD28 for T Cell Expansion and Activation | Thermo Fischer Scientific | Cat#11131D |
| D-Luciferin | Goldbio | Cat#LUCK-100 |
| Isoproterenol | Sigma-Aldrich | Cat#I6504 |
| **Critical commercial assays** | | |
| iScript cDNA Synthesis Kit | Bio-Rad | Cat#1708890 |
| RNeasy Plus Kit | Qiagen | Cat#74134 |
| iTaq Universal SYBR Green Supermix | Bio-Rad | Cat#1725121 |
| **Deposited Data** | | |
| CD8+ T-Cell RNA-seq | This paper; GEO | GSE192679 |
| **Experimental Models: Cell Lines** | | |
| Human: HEK293T | ATCC | Cat#CRL-3216 |
| Human: HEK293T β-arrestin 1/2 Knock Out | Asuka Inoue | ^1^ |
| Human: CD8+ T-Cells, Negatively Selected | Precision for Medicine | N/A |
| **Experimental Models: Organisms/strains** | | |
| C57BL/6 Female Mice | Charles River | Cat#C57BL/6NCrl |
| **Oligonucleotides** | | |
| CXCR3 fwd primer GCCATGGTCCTTGAGGTGAG | Sigma-Aldrich | N/A |
| CXCR3 rev primer GGAGGTACAGCACGAGTCAC | Sigma-Aldrich | N/A |
| 18s fwd primer GTAACCCGTTGAACCCCATT | Sigma-Aldrich | N/A |
| 18s rev primer CCATCCAATCGGTAGTAGCG | Sigma-Aldrich | N/A |
| **Recombinant DNA** | | |
| pcDNA3.1_CXCR3 | Rajagopal Lab | N/A |
| pcDNA3.1_CXCR3-RLuc2 | Rajagopal Lab | N/A |
| pcDNA3.1_Myrpalm-mVenus | Rajagopal Lab ^2^ | N/A |
| pcDNA3.1_2x-Fyve-mvenus | Rajagopal Lab ^2^ | N/A |
| β-arrestin 1 | Lefkowitz Lab | N/A |
| β-arrestin 2 | Lefkowitz Lab | N/A |
| pcDNA3.1_Mas-KB-1753-Nluc | Garcia-Marcos Lab (Maziarz et al. 2020) | N/A |
| pcDNA3.1_2xFyve-KB-1753-nLuc | This work | N/A |
| pcDNA3.1_Gαi-mVenus | Rajagopal Lab | N/A |
| Gαi1-LgBit | ^3^ | N/A |
| pcDNA3.1_2xFyve-SmBit | This work | N/A |
| NLuc-EPAC-VV | Martemyanov Lab ^4^ | N/A |
| NLuc-EPAC-VV-NLS | This work | N/A |
| pBk-HA-1-DI-K44A | Lefkowitz Lab | N/A |
| SmBit-Barr2 | Rajagopal Lab | N/A |
| pcDNA3.1_2xFyve-LgBit | This work | N/A |
| pcDNA3.1_SmBit-Barr2-FlAsH 4 | This work | N/A |
| pcDNA3.1_SmBit-Barr2-FlAsH 5 | This work | N/A |
| pcDNA3.1_LgBit-CAAX | This work | N/A |
| pcDNA3.1_Cyto-EKAR BRET Biosensor | This work | N/A |
| pcDNA3.1_Nuc-EKAR BRET Biosensor | This work | N/A |
| pGL4.33[*luc*2P/SRE/Hygro] | Promega | Cat#E1340 |
| pGL4.34[*luc*2P/SRF-RE/Hygro] | Promega | Cat#PS087 |
| pcDNA3.1_CXCR3-mCerulean | This work | N/A |
| pcDNA3.1_CXCR3-GFP | This work | N/A |
| β-arrestin 2-RFP | Marc Caron Lab | N/A |
| **Software and algorithms** | | |
| GraphPad Prism | GraphPad Software | <https://www.graphpad.com/scientific-software/prism/> |
| ImageJ | ^5^ | <https://imagej.nih.gov/ij/> |
| Adobe Illustrator | Adobe | <https://www.adobe.com/> |
| Excel | Microsoft | <https://www.microsoft.com/en-us/microsoft-365/excel> |
| ImageLab | Bio-Rad | <https://www.bio-rad.com/en-us/product/image-lab-software> |
| BioRender | BioRender | <https://biorender.com/> |
| Gene Set Enrichment Analysis | ^6^ | <https://www.gsea-msigdb.org/gsea/index.jsp> |

**References**

1. Alvarez-Curto, E. et al. Targeted Elimination of G Proteins and Arrestins Defines Their Specific Contributions to Both Intensity and Duration of G Protein-coupled Receptor Signaling. *J Biol Chem* **291**, 27147-27159 (2016).

2. Smith, J.S. et al. C-X-C Motif Chemokine Receptor 3 Splice Variants Differentially Activate Beta-Arrestins to Regulate Downstream Signaling Pathways. *Mol Pharmacol* **92**, 136-150 (2017).

3. Inoue, A. et al. Illuminating G-Protein-Coupling Selectivity of GPCRs. *Cell* **177**, 1933-1947 e25 (2019).

4. Masuho, I. et al. Distinct profiles of functional discrimination among G proteins determine the actions of G protein-coupled receptors. *Sci Signal* **8**, ra123 (2015).

5. Schneider, C.A., Rasband, W.S. & Eliceiri, K.W. NIH Image to ImageJ: 25 years of image analysis. *Nat Methods* **9**, 671-5 (2012).

6. Subramanian, A. et al. Gene set enrichment analysis: a knowledge-based approach for interpreting genome-wide expression profiles. *Proc Natl Acad Sci U S A* **102**, 15545-50 (2005).
