# Supplementary figures and images for: Location bias contributes to functionally selective responses of biased CXCR3 agonists
Source: Nat Commun. 2022 Oct 4;13:5846. doi: 10.1038/s41467-022-33569-2 (PMC9532441; doi:10.1038/s41467-022-33569-2)

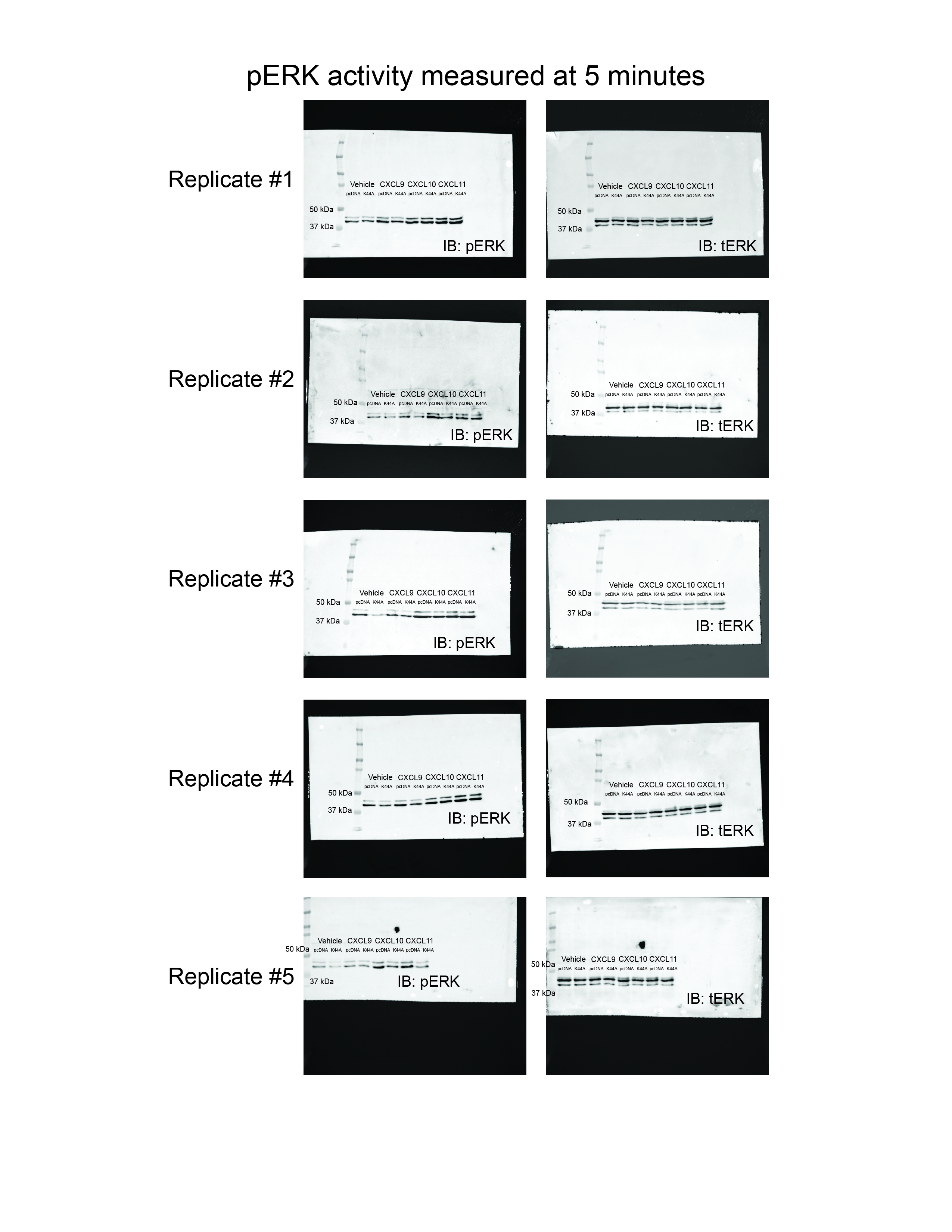

Supplement: Supplementary file 3 — Source Data [file 41467_2022_33569_MOESM3_ESM.zip › Source Data - Supplemental 1 - Unprocessed WB-01.jpg]

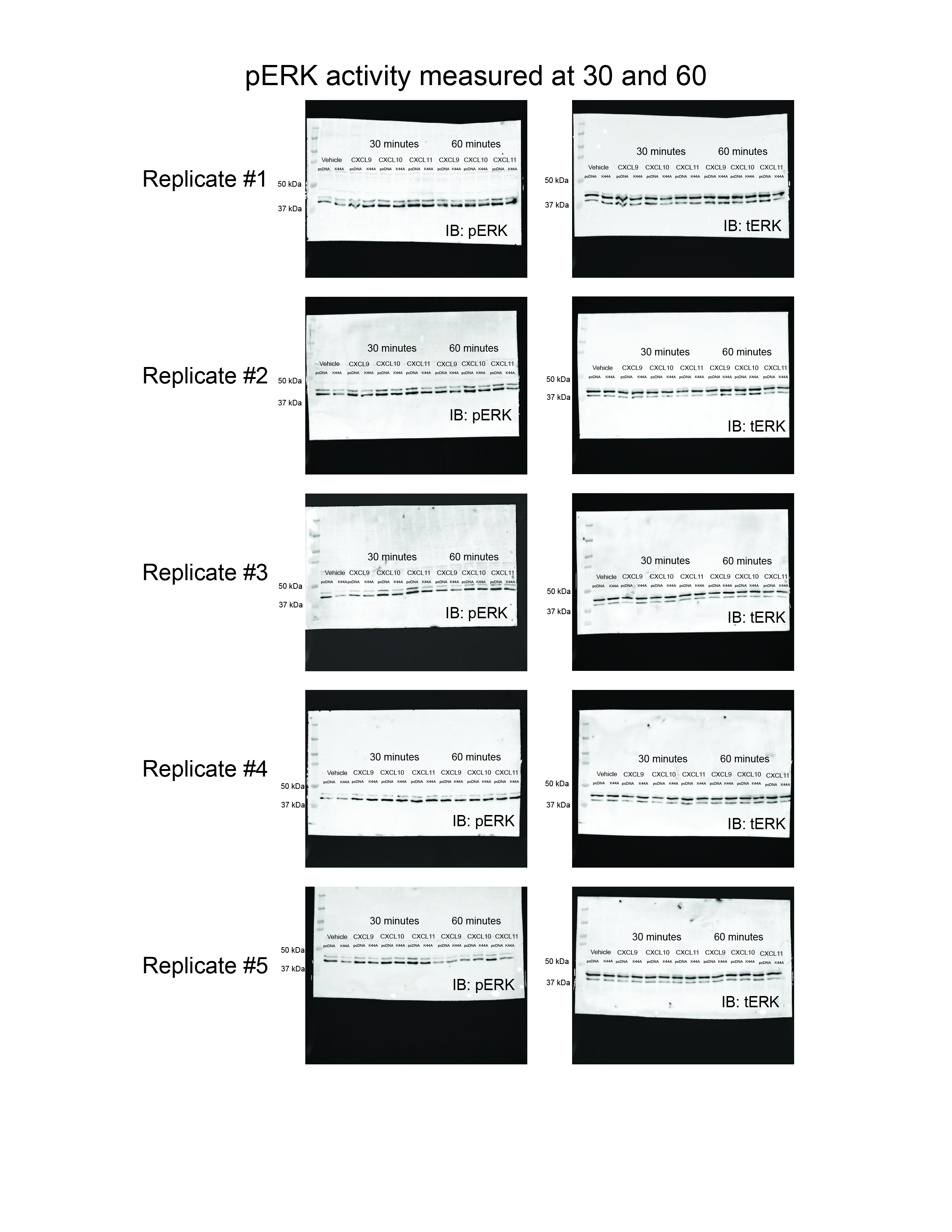

Supplement: Supplementary file 3 — Source Data [file 41467_2022_33569_MOESM3_ESM.zip › Source Data - Supplemental 2 - Unprocessed WB-02.jpg]
